# Effects of Bariatric Surgeries on Fetuin-A, Selenoprotein P, Angiopoietin-Like Protein 6, and Fibroblast Growth Factor 21 Concentration

**DOI:** 10.1155/2021/5527107

**Published:** 2021-08-06

**Authors:** Jakub Poloczek, Wojciech Kazura, Ewa Kwaśnicka, Janusz Gumprecht, Jerzy Jochem, Dominika Stygar

**Affiliations:** ^1^Department of Rehabilitation, 3rd Specialist Hospital in Rybnik, 44-200 Rybnik, Poland; ^2^Department of Internal Medicine, Diabetology, and Nephrology, Faculty of Medical Sciences in Zabrze, Medical University of Silesia, Katowice, Poland; ^3^Doctoral School of Medical University of Silesia, Department of Physiology, Faculty of Medical Sciences in Zabrze, 41-808 Zabrze, Poland; ^4^Pediatric Ward, Municipal Hospital in Żory, 44-240 Żory, Poland; ^5^Department of Physiology, Faculty of Medical Sciences in Zabrze, Medical University of Silesia, 41-808 Zabrze, Poland

## Abstract

Obesity is a civilization disease representing a global health problem. Excessive body weight significantly reduces the quality of life. It is also associated with the leading causes of death, including type 2 *diabetes mellitus*, cardiovascular diseases, and numerous types of cancer. The mainstay of therapy is a dietary treatment. However, in morbidly obese patients, dietary treatment is often insufficient. In these patients, the most effective procedure is bariatric surgery, but it is still difficult to predict its outcome and metabolic changes. Hepatokines are proteins secreted by hepatocytes. Many of them, including fetuin-A, selenoprotein P, angiopoietin-like protein 6, and fibroblast growth factor 21, have been linked to metabolic dysfunctions. In this context, hepatokines may prove helpful. This review investigates the possible changes in hepatokine profiles after selected bariatric surgery protocols. In this regard, *Roux-en-Y* gastric bypass is the most studied type of surgery. The overall analysis of published research identified fetuin-A as a potential marker of metabolic alternations in patients after bariatric surgery.

## 1. Introduction

Obesity is an inflammatory condition that is characterized by constant stimulation of the immune cells. Simultaneously, it is accompanied only by a slight increase in circulating proinflammatory factors and no evident clinical signs of inflammation [1]. Obesity is not only an aesthetic problem. It is also associated with a significant deterioration in the quality of life. It generates costs for the healthcare system and economic consequences for the individuals. It also contributes to many comorbidities such as cardiovascular diseases, respiratory problems, osteoarthritis, and certain types of cancer [2]. The mainstay of obesity management is nutritional treatment and therapy of secondary metabolic disorders, and the primary goal is to achieve weight loss and maintain it. However, in many patients, especially those with morbid obesity, such interventions are insufficient. For those patients who have failed to achieve and retain reduction of body weight by nonsurgical methods and for whom weight loss may deflect or refine adverse health effects, bariatric surgery may be the solution. Bariatric surgery procedures can be divided into restrictive procedures, which aim to significantly reduce the capacity of the gastric sac and consequently reduce food consumption, and malabsorptive procedures, which aim to limit the proper absorption of nutrients, and techniques combining both types of operations [3]. Bariatric surgery is the most efficient obesity treatment, providing permanent weight loss and as a side effect, reduction of insulin resistance and improvement of diabetes control [4, 5]. The surgical intervention induces alternations in signaling pathways and changes in the concentrations of different proteins, including these important for regulation of carbohydrate homeostasis [5]. Hepatokines are proteins produced by the liver [6, 7] that are mainly involved in the proper functioning of the carbohydrate metabolism [8–12]. Proteins of this group include, among others, selenoprotein P, fetuin-A, angiopoietin-like protein 6 (ANGPTL-6), and fibroblast growth factor 21 (FGF-21). Therefore, it is important to determine the effect of bariatric surgery on hepatokine concentrations.

## 2. Materials and Methods

PubMed and Google Scholar databases were searched using the following search criteria: “bariatric surgery” AND “hepatokines.” For this review, the four most known types of bariatric surgery: laparoscopic adjustable gastric banding (LAGB), sleeve gastrectomy (SG), *Roux-en-Y* gastric bypass (RYGB), and biliopancreatic diversion with duodenal switch (BPD-DS), were grouped based on the mechanism by which they cause weight loss and differentiated to restrictive (LAGB, SG) and combined (RYGB, BPD-DS) surgery protocols. Four hepatokines affected by bariatric surgery were selected: selenoprotein P, fetuin-A, angiopoietin-like protein 6 (ANGPTL-6), and fibroblast growth factor 21 (FGF-21). Additionally, the literature was analyzed to understand the potential impact of bariatric surgery and hepatokines on the resolution of obesity-related comorbidities.

### 2.1. Fetuin-A

Fetuin-A is a multifunctional hepatocyte-derived protein. It is directly involved in numerous metabolic pathways: calcium-phosphate homeostasis [13], immunological processes [14], and lipid and carbohydrate metabolism [15]. Human studies have demonstrated that it functions as a natural inhibitor of insulin receptor tyrosine kinase and impedes physiological insulin signaling in the liver and skeletal muscles, leading to the occurrence of insulin resistance [16], whereas, in the animal model, it acts as an endogenous ligand for Toll-like receptor 4 (TRL4) [17] and induces the decreased insulin sensitivity throughout stimulation of adipose tissue inflammation. In this context, fetuin-A concentration seems to be regulated by proinflammatory cytokines [18, 19].

Fetuin-A influence on the occurrence of metabolic disturbances was demonstrated in fetuin-null mice. The study showed that the lack of this hepatokine in mice decreased the risk of obesity and insulin resistance associated with aging processes [20]. The impact of high concentrations of fetuin-A on the development of disorders related to the metabolic syndrome was also confirmed by clinical studies, which reported that concentration of fetuin-A was positively associated with atherogenic lipid profile, BMI, waist circumference, and HOMA-IR score [21, 22]. In patients with impaired insulin sensitivity, high plasma levels of fetuin-A are associated with fat accumulation in the liver [23], whereas, in morbidly obese subjects, they may be correlated with fat accumulation in the visceral tissue [24]. Hence, increased levels of fetuin-A are a risk factor of insulin resistance and T2DM [25–28]. For all these reasons, some researchers maintain that fetuin-A is the most important protein of all hepatokines [7].

Fetuin-A has been the subject of numerous researches in the context of bariatric surgery. A pilot study from 2014 on 15 obese subjects after gastric bypass (GBP) (*n* = 8) and sleeve gastrectomy (SG) (*n* = 7) showed that levels of fetuin-A were significantly decreased in GBP patients three days after operation while the decline was not significant after SG [29]. The authors also found a statistically significant correlation between fetuin-A levels and HOMA-IR (*P* < 0.05). The authors concluded that the results suggested a direct relationship between insulin resistance and fetuin-A plasma concentration [29]. A study conducted by Brix et al. on 75 patients with morbid obesity showed that, 16 months after *Roux-en-Y* gastric bypass surgery, fetuin-A levels decreased and it was accompanied by a simultaneous weight loss [30]. The authors observed correlation between fetuin-A and ∆ insulin fasting (*r* = 0.710; *P* = 0.001) and HOMA-IR (*r* = 0.684; *P* = 0.001). Authors also suggested that a decrease in fetuin-A levels due to weight loss may play an important role in achieving a successful outcome after gastric bypass surgery [30]. Additionally, research on the obese Asian population by Yang et al. showed that twelve months after the surgical procedure, fetuin-A plasma levels also decreased significantly in all patients enrolled in the study (286.5 ± 53.7 vs. 258.8 ± 55.5 *μ*g/ml (*P* < 0.001)), regardless of the type of performed surgery [27]. Unfortunately, even though the research included 130 patients who underwent *Roux-en-Y* gastric bypass (RYGB) (*n* = 41), minigastric bypass (MGB) (*n* = 67), and sleeve gastrectomy (SG) (*n* = 22), the authors did not compare the effectiveness of different surgical methods among themselves, which is considered a limitation of this study. Also, it is worth noting that from 43 patients suffering from T2DM before the surgery, only five of them remained diabetic after a bariatric procedure [27]. Taking into consideration that glycated hemoglobin (HbA1c) exhibited a strong correlation both with pre- and postoperative circulating fetuin-A concentration, the authors of this research suggested that fetuin-A may play a role in glucose homeostasis in patients with severe obesity and suggested considering it a marker, even after the bariatric operation.

A prospective, longitudinal (12 months) study conducted by Huang et al. [31] compared the changes in fetuin-A levels after gastric bypass (GB) (*n* = 18) and sleeve gastrectomy (SG) (*n* = 16) in nonmorbidly obese patients with T2DM [31]. In patients after GB surgery, they found a significant positive relationship between fetuin-A and HbA1C concentration (*ρ* = 0.517, *P* = 0.033). Also, *Δ* fetuin-A positively correlated only with *Δ* BMI (*ρ* = 0.574, *P* = 0.031) and *Δ* waist-to-hip ratio (*ρ* = 0.538, *P* = 0.031). In patients after SG surgery, fetuin-A showed no significant correlation with any of the anthropometric and biochemical parameters assessed in the study. The authors also noted at the one-year follow-up that neither GB nor SG procedure had statistically significant temporal effects on fetuin-A plasma levels [31]. The lack of postoperative reduction of fetuin-A levels was inconsistent with earlier studies that showed a significant drop in fetuin-A levels after bariatric surgeries performed; however, these studies were conducted on morbidly obese patients [27, 29, 30]. Huang et al. suggested that lower initial BMI of patients enrolled in their study might have been a contributing factor to a nonsignificant reduction of fetuin-A concentration after the surgical procedure [31].

Although bariatric surgery lowers the concentration of circulating fetuin-A through different mechanisms, it appears that one of the most important factors is weight loss per se. This was shown by a study conducted on 36 obese children who took part in a one-year ambulatory intervention program consisting of physical exercise, nutrition education, and behavior therapy. Twenty-one children, who managed to substantially reduce body weight, showed a significant decrease in fetuin-A concentration (*P* = 0.029). Fifteen children, whose body weight did not alter significantly within the one year of lifestyle intervention, showed no changes in fetuin-A plasma concentration (*P* = 0.433) [32]. The importance of supportive interventions, such as changing the nutritional model, in the context of therapeutic success after bariatric surgery was shown also in an animal model by Stygar et al. [33]. Fifty-six rats were randomly assigned to one of the dietary regimens: a high-fat diet or a balanced control diet. Eight weeks later, animals underwent either duodenal-jejunal omega switch (DJOS) surgery or control (SHAM) surgery. After the operation, in each group, half of the randomly selected animals had their diet changed for the next eight weeks. The results showed that DJOS contributed to a substantial reduction in fetuin-A plasma concentration and the decrease in the relative expression of the fetuin-A gene in the liver when compared to a control procedure. The study also showed that in DJOS-operated rats, the introduction of a high-fat diet at any point of the experiment led to a significant increase in fetuin-A concentration when compared to rats fed on a balanced diet throughout the study. The introduction of a high-fat diet resulted also in the upregulation of hepatic Ahsg gene relative expression [33]. The study proved that maintaining appropriate eating patterns can result in significant improvements in the metabolic profile and may increase the effectiveness of the bariatric procedure.

### 2.2. Selenoprotein P

Selenoprotein P is a secretory glycoprotein produced and released mainly by the liver [34]. It acts as a selenium transporter to the peripheral tissues [35] and directly modulates inflammatory processes induced by oxidative stress [36]. Selenoprotein P is a protein with antioxidant properties because it stimulates glutathione peroxidase expression [37–39]. As a selenium transporter, it regulates antioxidant enzymes' activities and inflammatory responses [40].

The links between selenoprotein P and metabolic diseases are currently of interest to many researchers, and the state of knowledge on this topic constantly increases. Research on the effects of surgical procedures on selenoprotein P concentration is very scarce. So far, only one study has assessed the impact of bariatric surgery on selenoprotein P plasma level. Lim et al. studied ten patients who underwent RYGB surgery to treat obesity or T2DM [41]. They observed a linear increase in selenoprotein P concentration over nine months after RYGB. The changes negatively correlated with insulin resistance expressed by *Δ* HOMA-IR (*r* = −0.770, *P* < 0.01) [41], which proved the beneficial effect of RYGB on carbohydrate metabolism.

The relationship between selenoprotein P levels and the occurrence of many metabolic disorders is well-documented. Its high plasma concentration is associated with dysregulated glucose metabolism, nonalcoholic fatty liver disease, and enhanced atherogenesis leading to cardiovascular diseases [42–44]. An animal-based experiment by Misu et al. showed that the increase of selenoprotein P level may induce hyperglycemia and insulin resistance [9]. They also confirmed that glucose and palmitate take part in the upregulation of selenoprotein P expression in hepatocytes, while Jung et al. showed that insulin reduces its expression [9, 45]. Also, selenoprotein P knockout mice showed enhanced glucose tolerance, depleted plasma insulin concentration, and ameliorated signaling of the insulin pathway in liver and skeletal muscles [9]. Nonetheless, some studies show that reduced plasma level of selenoprotein P is not beneficial to health [46–49]. Studies on animals and humans showed that selenoprotein concentrations outside the norm, both below and above their physiological norm, may correlate with T2DM development [50, 51]. The mechanism of this phenomenon is multifactorial. It may depend on the stage and advancement of the disease. It could also be explained by the imbalance between oxidative and reductive stressors, and fluctuating expression of antioxidative enzymes such as glutathione peroxidase-1 [50, 51]. However, the results of the research are inconsistent, and further investigation is needed.

### 2.3. Angiopoietin-Like Protein 6

Angiopoietin-like protein 6 (ANGPTL-6) is a circulating protein expressed mostly in the liver [52]. Recent studies have shown that this hepatokine has a positive effect on metabolism as it counteracts obesity and obesity-associated insulin resistance, promotes angiogenesis, and takes part in the healing process by supporting the proliferation of keratinocytes [10, 53, 54].

The relationship between ANGPTL-6 and glucose metabolism on the molecular level has been investigated in animal studies. The studies have proven that ANGPTL-6 suppresses gluconeogenesis in hepatocytes and improves insulin signaling in skeletal muscles [55]. Animal-based research has also shown that deficiency of ANGPTL-6 may result in obesity, decreased insulin sensitivity, and weakened systemic energy expenditure, despite following a balanced, normocaloric diet [10]. On the contrary, mice with targeted ANGPTL-6 activation showed a 2.5-fold increase in serum ANGPTL-6 concentration, increased insulin sensitivity, and were protected from diet-induced obesity [10]. However, in humans, serum ANGPTL6 levels correlate with the metabolic syndrome indicators such as BMI, blood glucose, and HOMA-IR [56]. Some studies even suggest that patients with T2DM or metabolic syndrome have higher ANGPTL-6 plasma levels [57–60], which seem to contradict the earlier assumptions regarding the role of ANGPTL-6 in obesity and insulin resistance prevention. Thus, further researches in this area are warranted.

Currently, only a few researchers have explored changes in ANGPTL-6 concentration after bariatric surgery. The above-mentioned study by Lim et al. conducted on ten obese patients subjected to RYGB revealed that serum ANGPTL-6 concentration significantly decreased one month after the operation and its low level was maintained nine months after the surgery [41]. ANGPTL-6 serum concentrations significantly correlated with LDL cholesterol and fasting insulin values, while changes in ANGPTL-6 levels corresponded to changes in total cholesterol and LDL cholesterol levels one month after operation [41]. Another experiment conducted by Cinkajzlova et al. on 64 morbidly obese adults (23 nondiabetic and 40 diabetic) has shown that serum ANGPTL-6 levels of obese patients did not differ significantly from the control group regardless of diabetes presence [56]. These results contradict previous [57–59] and more current reports [60] mentioned above. Also, following a very-low-calorie diet for three weeks resulted in a decrease of ANGPTL-6 concentration, but only in diabetic patients. In patients with T2DM, who underwent a bariatric operation (gastric plication, *n* = 10; gastric banding, *n* = 2; gastric bypass, *n* = 1), ANGPTL-6 serum levels did not differ relevantly from the control group in a long-term follow-up. However, the surgical procedure led to a temporary and transient elevation of ANGPTL-6 concentration one month after operation [56].

### 2.4. Fibroblast Growth Factor 21 (FGF-21)

Fibroblast growth factor 21 (FGF-21) is a protein secreted by the liver [6] and induced in a fasting state [61]. Animal- and human-based studies have shown that FGF-21 has a beneficial effect on glucose metabolism, as it may induce glucose uptake and improve insulin resistance [11, 12]. Moreover, FGF21 is considered capable of regulating food preferences and downregulating carbohydrate intake [62]. It also lessens the likelihood of obesity development in rodents [63, 64]. FGF-21 plasma levels are correlated with BMI and insulin sensitivity [11, 65, 66], and numerous studies have shown a higher concentration of FGF-21 in obese or T2DM subjects [11, 28, 65–67]. Also, it has been suggested that components of the metabolic syndrome may cause the state of resistance to FGF-21 [68].

There are only a few studies based on animal models, which concentrated on bariatric surgery and hepatokines. In diabetic rats, ileal transposition (IT) surgery significantly depleted FGF-21 plasma concentration but elevated the expression of the fibroblast growth factor 1 (FGF-1) gene and FGF-1 concentration and FGF-21 concentration in visceral adipose tissue and the liver when compared to rats that underwent control surgery. The changes were accompanied by a bodyweight loss, daily food intake decrease, and glucose and lipid metabolism improvement [69]. The research evaluating the effects of DJOS surgery and various nutritional models on the FGF-21 plasma concentration showed that the effect of bariatric surgery was associated with a significant reduction of FGF-21 concentration 8 weeks after the operation, when compared to sham surgery, in rats that received a high-fat diet during the experiment [33]. Concomitantly, in animals subjected to the DJOS surgery but having different dietary protocols, no differences in the level of FGF-21 were observed depending on the nutritional model. However, when the HFD diet was used before DJOS surgery, the hepatic FGF-21 gene expression was lower after the surgery [33]. Similar results were achieved by Liu et al. who subjected rats with diet-induced diabetes to duodenal-jejunal bypass surgery (DJB) and sleeve gastrectomy (SG) [70]. When compared to SHAM-operated rats, both DJB- and SG-operated rats showed improved glucose homeostasis and insulin sensitivity expressed through a reduction in fasting blood glucose, alleviation of oral glucose tolerance test and insulin tolerance test profiles, and depletion of HOMA-IR value. Moreover, both bariatric operations contributed to the improved lipid profile in the rats' plasma, lipid accumulation in the liver, and the reduced size of adipocytes. After bariatric surgeries, both plasma concentration of FGF-21 and its hepatic expression were significantly reduced in comparison to rats after control surgery. The authors noted that both DJB and SG decreased the level of circulating FGF-21 and it was lower than that found in animals fed with a control diet, but SG was more effective in this regard [70]. Another noteworthy study, by Morrison et al., investigated the influence of bariatrics on changes in metabolism in FGF-deficient (FGF-21-/-) and wild-type (WT) mice [71]. The study revealed lower FGF-21 plasma levels and hepatic mRNA expression of the FGF-21 gene in RYGB-operated mice when comparing with SHAM-operated animals six weeks after surgery. However, the hepatokine concentration was significantly higher in RYGB-operated mice when compared to calorie-restricted mice with similar weight loss [71]. Interestingly, the lack of FGF-21 receptor affected neither changes in body weight, food intake, and energy expenditure nor improvement of glycemic control and insulin sensitivity after surgery in comparison with WT mice. The authors concluded that signaling of FGF-21 is not a pivotal single component required by bariatric surgery to exert metabolic effects but may play a minor, supporting role [71].

Studies on humans, in turn, do not provide a clear picture of changes in FGF-21 levels. The effect of bariatric surgery on the FGF-21 level has been studied by several research groups. Vienberg et al.'s study, performed on eight obese nondiabetic humans that underwent RYGB, showed that 14 days after operation fasting plasma FGF-21 concentration did not differ significantly from its concentration before surgery (262 ± 71 vs. 411 ± 119 pg/ml, *P* = 0.13) [72]. However, in patients who underwent RYGB surgery, the concentration of FGF-21 increased significantly after oral glucose load, but only in the absence of protein. Therefore, the authors rejected the thesis that the level of FGF21 could have been regulated by the concentration of glucagon [72], which was postulated in earlier studies [73]. Woelnerhanssen et al., in their study including 23 morbidly obese nondiabetic patients, randomly subjected to laparoscopic RYGB (*n* = 12) or laparoscopic sleeve gastrectomy LSG (*n* = 11), also showed no statistically significant differences in the FGF-21 serum levels after these two types of surgery during 1-year observation [74]. Bužga et al. investigating the long-term impact of laparoscopic greater curvature plication (LGCP) obtained similar results to the aforementioned study. They observed only a slight, but not significant, decrease in FGF-21 levels in 52 obese subjects one year after the surgery [75]. Distinct results were reported by Jansen et al. who performed a study on 35 morbidly obese adults subjected to laparoscopic RYGB [76]. They reported that despite the initial elevated level of circulating FGF-21, compared to healthy volunteers in the control group, an even higher and statistically significant increase in FGF-21 concentration was observed 3 months after the surgery [76]. A similar observation on RYGB-operated patients was done by Lips et al. who conducted a study on morbidly obese nondiabetic and diabetic females subjected to various weight loss interventions, such as the implementation of a very-low-calorie diet (VLCD) (*n* = 12), gastric banding surgery (*n* = 11), or RYGB surgery (*n* = 31) [67]. They assessed FGF-21 levels before, 3 weeks, and 3 months after the intervention. The VLCD resulted in a permanent reduction of FGF-21 concentration [67]. Gastric banding did not change FGF-21 concentration 3 weeks after the procedure, but 3 months after it, the FGF-21 plasma level decreased (*P* < 0.05). RYGB, by contrast, resulted in increased FGF-21 plasma levels 3 weeks after the operation regardless of diabetes status; nonetheless, 3 months after the procedure, the significantly elevated FGF-21 levels remained only in nondiabetic patients. Also, the concentration of FGF-21 was significantly higher in the RYGB group, when compared to the VLCD, or the GB group (*P* < 0.0001) [67]. Similar results were presented by Gómez-Ambrosi et al. who conducted a study on 137 obese subjects, from which 114 were qualified for *Roux-en-Y* gastric bypass (RYGB, *n* = 66), sleeve gastrectomy (SG, *n* = 20), and dietary intervention (*n* = 28) [77]. Before the treatment, all obese patients, regardless of the presence of carbohydrate metabolism disorders, had statistically significant higher fasting FGF-21 concentration when comparing with lean subjects from the control group. During one year of the study, the authors observed a significant decrease of FGF-21 levels after the dietary intervention and after SG when comparing with the initial state. RYGB surgery, however, had no significant impact in this regard [77]. Haluzíková et al. investigated the influence of laparoscopic SG on FGF-19 and FGF-21 levels in 17 obese females [78]. They noted that FGF-21 concentration was significantly reduced 12 months after the procedure and stayed low two years after it [78]. The latest study from 2017 by Fjeldborg et al. on 31 obese patients (including 11 subjects with T2DM) revealed that there was no difference in FGF-21 levels between diabetic and nondiabetic subjects before RYGB operation, but obese patients had a significantly higher concentration of FGF-21 when compared with lean subjects [65]. They observed that RYGB did not cause significant changes in FGF-21 serum levels. Twelve months after the surgery, a significant decline in FGF-21 concentration was observed only in a group of patients with high basic FGF-21 levels [65].

Taking into account the dispersion of the results in many studies, it should be emphasized that the unclear picture of FGF-21 changes may result from its wide concentration range, as found by Kharitonenkov et al. in a control group of healthy people (range 21-5300 pg/ml) [79]. Nevertheless, scientists started to exploit FGF-21 as a novel therapeutic drug. Research by Gaich et al. on obese diabetic patients, who received exogenous FGF-21 in a randomized, placebo-controlled, double-blind proof-of-concept trial revealed improvement of lipid profile, body weight, and fasting insulin levels [80]. Pharmacological alternatives for bariatric surgical procedures are also being developed. They aim to help avoid periprocedural complications and enable the treatment of patients with contraindications to traditional surgery while providing comparable therapeutic effects to surgical treatment. The GLP-1-FGF-21 fusion protein that might support or even replace surgical treatment in the future could become such a drug for patients struggling with obesity [81]. However, further research is needed in this regard.

## 3. Summary

*Roux-en-Y* gastric bypass was the most studied procedure in the context of hepatokines. To a lesser extent, such operations as sleeve gastrectomy, gastric bypass surgery, or laparoscopic greater curvature plication were also taken into account. However, all of the discussed studies were carried out on a relatively small number of patients (from 8 to 137), whereas the observation time of patients is easier to compare, as follow-ups varied from 3 days to 2 years after surgery. Among the discussed hepatokines, only two of them have been thoroughly investigated. Regardless of the number of patients and the time of observation, numerous clinical trials confirmed the practical usefulness of fetuin-A in patients after bariatric surgery. In our opinion, it can play the role of a marker of the alternations in insulin sensitivity of the cells. As for FGF-21, research confirmed only a strong relationship between obesity and metabolic disturbances. The results, however, were very heterogeneous, which impedes straightforward interpretation. There are not enough studies on the remaining hepatokines, ANGPTL-6 and selenoprotein P, to conclude if they may be useful for monitoring the treatment outcomes and assessing the prognosis. The issue requires further, thorough investigation.

## Data Availability

The data is available after contact with the corresponding author.
